# Maize Prolamins Could Induce a Gluten-Like Cellular Immune Response in Some Celiac Disease Patients

**DOI:** 10.3390/nu5104174

**Published:** 2013-10-21

**Authors:** Juan P. Ortiz-Sánchez, Francisco Cabrera-Chávez, Ana M. Calderón de la Barca

**Affiliations:** 1 Department of Nutrition, Research Center for Food and Development (CIAD, A.C.), Carr. La Victoria, Km. 0.6, Hermosillo, Sonora 83304, Mexico; E-Mail: juanpedroo@gmail.com; 2 Nutrition Sciences and Gastronomy Unit, University of Sinaloa, Culiacan, Sinaloa 80019, Mexico; E-Mail: fcabrera@uas.edu.mx

**Keywords:** celiac disease, cellular immune response, maize prolamins, zeins

## Abstract

Celiac disease (CD) is an autoimmune-mediated enteropathy triggered by dietary gluten in genetically prone individuals. The current treatment for CD is a strict lifelong gluten-free diet. However, in some CD patients following a strict gluten-free diet, the symptoms do not remit. These cases may be refractory CD or due to gluten contamination; however, the lack of response could be related to other dietary ingredients, such as maize, which is one of the most common alternatives to wheat used in the gluten-free diet. In some CD patients, as a rare event, peptides from maize prolamins could induce a celiac-like immune response by similar or alternative pathogenic mechanisms to those used by wheat gluten peptides. This is supported by several shared features between wheat and maize prolamins and by some experimental results. Given that gluten peptides induce an immune response of the intestinal mucosa both *in vivo* and *in vitro*, peptides from maize prolamins could also be tested to determine whether they also induce a cellular immune response. Hypothetically, maize prolamins could be harmful for a very limited subgroup of CD patients, especially those that are non-responsive, and if it is confirmed, they should follow, in addition to a gluten-free, a maize-free diet.

## 1. Introduction

Celiac disease (CD) is an immune-mediated enteropathy triggered by dietary wheat, rye and barley gluten (water-insoluble proteins) in genetically predisposed individuals [1]. Characteristic features of CD involve crypt hyperplasia, intra-epithelial lymphocytosis and villus atrophy of the intestinal mucosa. These injuries affect intestinal function and nutrient absorption, which can cause a variety of gastrointestinal and extra-intestinal symptoms [2].

Intestinal mucosa damage in CD patients begins with an innate response that leads to a cellular immune response [3]. First, prolamin peptides from gluten, which are resistant to human digestion, interact with a chemokine receptor, inducing zonulin release and a subsequent tight junction disassembly [4]. Then, the damaged barrier allows the arrival of gliadin peptides to the lamina propria, where tissue transglutaminase (tTG) deamidates specific glutamine residues to confer an overall negative charge. These peptides are bound to the human leucocyte antigen (HLA) DQ2 or DQ8 molecules, in antigen presenting cells, which present them to T-cells to develop the full immune response required for CD [5]. In addition to gluten peptides, self tTG is presented to T-cells, which triggers an auto-immune response. Therefore, CD is considered an autoimmune disease.

CD symptoms disappear in the majority of patients after dietary gluten withdrawal; however, in some patients, the symptoms are still present even after they adopt a strict gluten-free diet [6]. This is due to either refractory CD or to the presence of gluten as a contaminant or as a non-declared additive in foods [7]. Additionally, the lack of response to dietary gluten withdrawal in a very limited subgroup of patients, could be due to other dietary proteins present in the gluten-free diet, such as those from maize, which is a common alternative ingredient used in gluten-free diets.

It has been demonstrated that zeins, the maize prolamins, are able to induce an inflammatory response through contact with the mucosa in some CD patients [8]. Furthermore, IgA antibodies from some CD patients can recognize zeins [9], even after lime and/or enzymatic treatments [10]. Perhaps, in active CD, peptides derived from zeins could exacerbate the immune response in the intestinal mucosa, because they have sequence characteristics and/or electronegative residues that resemble gluten peptides.

## 2. Supporting Experimental Results

Table 1 summarizes the similarities between maize prolamin peptides and wheat celiac-toxic gluten peptides that are involved in the pathogenesis of celiac disease. These results support the hypothesis that peptides from zeins that are resistant to human digestion are able to induce a celiac-like immune response in some CD patients by a similar mechanism to that triggered by wheat gluten peptides.

### 2.1. Incomplete Protein Digestion

Pepsin and trypsin, the main peptidases of the intestinal tract, cannot completely digest wheat gluten, because they are unable to cut its 15% proline-containing polypeptides [11,12]. The result is the release of peptides larger than nine amino acids, which are capable of eliciting innate and adaptive immune responses [13]. The proline content of zeins is also high (9%) and, although zeins contain bonds that pepsin can cut, they also contain cysteine residues with disulfide bonds that obstruct digestion by pepsin [14]. All together, the ability of trypsin to digest zeins is low due to their low number of cleavage sites, low solubility [15] and secondary conformation [16].

**Table 1:** Similarities between maize prolamin peptides and wheat celiac-toxic gluten peptides that are involved in the pathogenesis of celiac disease (CD). NO: nitric oxide; NOS: nitric oxide synthase; HLA-DQ2 or DQ8:human leucocyte antigen molecules; IFN-γ: interferon gamma.

Step in CD Pathogenesis		Characteristics of Celiac-Toxic Peptides from Wheat Gluten		Characteristics of Maize Prolamins That Could be Inducers for CD	
Incomplete protein digestion		Gastrointestinal peptidases do not digest the proline-rich wheat gluten polypeptides completely, which releases peptides larger than nine amino acids [11,12].		Digestion of zeins is poor due to relatively high concentrations of glutamine, proline and cysteine residues [14,15,16].	
Innate immune response		Increased levels of NO were produced by challenged granulocytes and NOS expression was increased in enterocytes from CD patients’ small intestine biopsies [17,18].		Proteins from maize caused granulocyte activation in a rectal challenge in six out of 13 CD patients tested [8].	
Adaptive immune response: deamidation of peptides by tTG		Gluten peptides deamidated by tTG in the lamina propria contain negative charges [19,20,21].		Maize prolamins deamidated by TG *in vitro* were better recognized than native ones by IgA from some CD patients’ sera [22].	
Adaptive response: increased affinity of HLA-DQ2/DQ8 on antigen presenting cells to bind peptides		HLA-DQ2 prefers negatively charged amino acids from gluten peptides at the p4, p6 or p7 positions in the peptide, while HLA-DQ8 prefers them at positions p1 or p9 [20].		Peptides from digested maize prolamins have glutamine at positions p1 and p9 that can be deamidated by tTG and bind to HLA-DQ8 [23,24]. Other peptides can be bound by HLA-DQ2 [10].	
Adaptive response: processing and presentation of peptides		After processing, the deamidated gluten peptides are presented to T-cells. Then, B-cells are induced to proliferate and produce antibodies [25].		T-cells from the intestine of one out of seven CD patients stimulated by maize prolamins and teff produced low IFN-γ as compared to wheat, but higher than control and other non-wheat grains [26]. Additionally, IgA antibodies against maize prolamins were detected in several CD patients [10,27].	
Adaptive response: role of antibodies against dietary prolamins		Roles of tTG-specific antibodies induced by gluten in CD patients could be: inhibiting epithelial cell differentiation and inducing their proliferation, increasing epithelial and blood vessel permeability and affecting angiogenesis [28].		Although the levels of antibodies against gluten decrease in some CD patients following a gluten-free diet, antibodies against maize prolamins remained high until both gluten and maize were avoided [29,30].	
Adaptive response: activation of T-cells		Activated T-cells drive the inflammatory response that leads to the development of the characteristic celiac lesions and the symptoms [31]. T-cells induce damage mostly by IFN-γ production [32].		Neither the intestinal lesions nor the CD symptoms were alleviated with a gluten-free diet when maize was still eaten [29].	

### 2.2. The Inflammatory Process

Nitric oxide (NO) production is involved in the innate inflammatory response mediated by macrophages in CD, and it has been detected in cultured gluten-challenged small intestine biopsies [17]. Additionally, there is an elevated expression of mRNA encoding the major inducible isoform of NO synthase II (iNOS) in untreated CD patients [18]. After rectal wheat gluten challenge in CD patients, granulocyte activation precedes NO production. Furthermore, some patients have been found to display signs of a similar inflammatory reaction after challenge with maize prolamins [8].

### 2.3. Deamidation of the Peptides

Gluten peptides are transported across the epithelial barrier to the lamina propria, where tTG changes the glutamine residues to glutamic acid. Antigen-presenting cells then process these negatively charged peptides and increase their affinity for the major histocompatibility complex (MHC) class II molecules, HLA-DQ2 and HLA-DQ8. These immunogenic peptide fragments can stimulate HLA-DQ2- and HLA-DQ8-restricted T-cells and trigger an adaptive response in the lamina propria [19,20,21]. Maize prolamins likely are also deamidated by tTG, because IgA from CD patients was more immunoreactive against maize prolamins extracted from maize bread, treated with microbial transglutaminase, than against maize prolamins from untreated bread [22].

### 2.4. Affinity of HLA/DQ8 Molecules to Bind Peptides

Adaptive responses to gluten initiate when dendritic cells phagocytose gliadin peptides and present them to undifferentiated T helper cells, whose activation is crucial for the development of CD. Peptide deamidation by tTG increases the affinity of HLA-DQ2/DQ8 for these peptides. HLA-DQ2 has an affinity for negatively charged amino acids at the p4, p6 or p7 positions in the peptide, while HLA-DQ8 has an affinity for those residues at positions p1 and p9 [23]. The primary amino acid sequences of maize zeins can fit into these HLA binding sites once they are deamidated. Through *in silico* analysis, Darewicz *et al*. [24] identified a high degree of homology between two zein peptides and the celiac-toxic peptides from prolamins found in wheat, barley and rye (gliadins, hordeins and secalins, respectively). Moreover, we have identified a peptide sequence (α-zein 58–91) that is resistant to complete digestion and which has characteristics that would allow it to bind to HLA-DQ8 [10]. In addition to this peptide, Table 2 provides the sequence of a 33-mer (α2-gliadin 56–88) peptide that is a potent T-cell stimulator [19].

**Table 2:** Theoretical peptide sequences that bind to HLA-DQ2/DQ8 molecules. After deamidation by tTG [33], glutamine residues (underlined) became glutamic acid, which is an electronegative residue that binds to p4 and 6 in HLA-DQ2 and p1 and 9 in HLA-DQ8.

Food	Peptide	Sequence	Affinity	Reference
Wheat	α-Gliadin	LQLQPFPQPQLPYPQPQLPYPQPQLPYPQPQPF	HLA-DQ2	[19]
Wheat	α Gliadin	LQLQPFPQPQLPYPQPQLPYPQPQLPYPQPQPF	HLA-DQ8	[19]
Maize	α-Zein	LQQAIAASNIPLSPLLFQQSPALSLVQSLVQTIR	HLA-DQ8	[10]

### 2.5. Processing and Presentation of Peptides

Peptides of gliadin are deamidated by tTG, phagocytosed, processed and transported to the cell surface in dendritic cells via MHC class II molecules. Subsequently, the peptides are presented to infiltrated T helper cells that recognize deamidated peptides and trigger the proliferation of specific B-cells and the production of IgA anti-gliadin and anti-transglutaminase antibodies [25]. Some celiac patients contain B-cells that produce anti-maize prolamin IgA antibodies that do not cross-react with anti-wheat prolamins [10,27].

### 2.6. Role of Antibodies

After the DQ2-/DQ8-dependent activation of CD4+ T-cells, B-cells are stimulated and produce auto-antibodies. These auto-antibodies in the intestinal lumen could be involved in disease pathogenesis in various ways. For instance, they could be involved in inhibiting epithelial cell differentiation, augmenting epithelial cell proliferation, increasing epithelial and blood vessel permeability and affecting angiogenesis [28]. In some CD patients on a gluten-free diet, including maize-based foods, the anti-gliadin and anti-tTG antibody titers diminished, but the symptoms persisted [29,30]. Total symptom remission in these cases was achieved only with a gluten- and maize-free diet [30]. It is possible that partial production of anti-tTG antibodies, in addition to anti-zein antibodies, continued to affect the intestinal mucosa when dietary maize was present.

### 2.7. Activation of T-Cells

The activation of gliadin-reactive CD4+ T-cells results in the production of cytokines that drive an inflammatory response, which leads to the development of the characteristic CD lesions and symptoms [31]. Gluten-specific T-cells induce tissue damage mostly by the production of interferon (IFN)-γ [32]. There is some evidence of T-cells being simulated by maize prolamins: intestinal T-cells cultured from CD patients were challenged with maize prolamins *in vitro*, and T-cells from one out of seven samples produced IFN-γ as a result of T-cell stimulation [26]. Although this patient response was not specific, maize and teff peptides produced higher levels of IFN-γ (145.6 and 154.4 pg/mL, respectively) than the negative control (10.9 pg/mL) and others “non-toxic” grains (≈110 pg/mL).

Dietary gluten withdrawal has been demonstrated to induce mucosal recovery and the disappearance of CD symptoms. Nevertheless, some patients on gluten-free diet have forms of CD that do not respond to this diet. This could be due to a higher sensibility of these patients to “gluten-free” foods that still contain some traces of gluten [34] or to the presence of other cereal prolamins, such as those in maize in a very limited subgroup of CD patients.

## 3. Potential Links between Zeins and CD

Based on the similarities between wheat and maize prolamins discussed above, we can infer that the innate and adaptive responses to zeins would be similar to the response against gliadins in CD patients. Nevertheless, it is necessary to identify whether zeins contain immunodominant and minor epitopes similar to those found in gliadins after proteolysis. Some authors have found that there is no effect on T-cell activation or pro-inflammatory cytokine secretion when CD patient biopsies were treated with whole pepsin-trypsin digested prolamins from maize [26,35]. Therefore, there is a need to evaluate the effect of isolated immunogenic peptides from maize prolamins, which can be obtained by *in silico* analysis [10].

The evaluation of the response of immune cells to gliadins includes the increased expression of surface receptors and the production of different cytokines for both tissue and immune cells. Some of these receptors include HLA-DR (human leucocyte antigen), CD54 or ICAM-1 (intercellular adhesion molecule), CD3 (in mature T-cells), CD25 (interleukin-2 receptor) and CD69 (in activated T-cells and natural killer cells) [36,37]. Cytokines that would be produced include interferon gamma, interleukins (IL) 2 and 15 and zonulin [13,38,39,40]. To evaluate the immune response, an analysis of the protein expression of these markers can be performed after CD patient biopsies are challenged with zein peptides. These *ex vivo* digested-peptide challenge analyses are considered useful tools to evaluate the safety of non-gluten prolamins in a gluten-free diet [26,40].

There is evidence that after a short gluten challenge in treated CD patients, gluten-specific T-cells are present in peripheral blood [41,42,43,44]. After this *in vivo* challenge, peripheral blood mononuclear cells can be isolated and activated with gluten peptides for quantitative detection of pro-inflammatory cytokines and direct detection of HLA-DQ2 tetramer specific for gliadins. For both cytokine measurements and the detection of an immune response, these techniques would be very useful in the evaluation of the effect of maize prolamins on the immune response in CD patients.

## 4. Conclusions

Although reaction to maize prolamins in CD patients appears to be a rare event, the confirmation that they play a role in the pathogenesis of CD will be useful information for the follow-up of some non-responsive celiac patients. It is estimated that approximately 10% to 18% of these cases are refractory CD, which represents a more severe CD, with a clear malignity and a less favorable prognosis [7]. Therefore, it is important to assess these clinical cases, because uncontrolled CD can lead to several malabsorption problems, osteoporosis and other autoimmune diseases [45].

Maize is one of the most commonly consumed grains in the gluten-free diet. Despite the low content of zeins in maize-containing foods compared with that of gliadins in wheat-containing foods, maize could be responsible for persistent mucosal damage in a very limited subgroup of CD patients. If our hypothesis is proven, zeins could be classified as harmful for some CD patients, especially those showing a poor response to a gluten-free diet.
